# Interhospital Transportation of Pediatric Patients Undergoing Venovenous Extracorporeal Membrane Oxygenation (VV ECMO) Support—A 3-Year Regional Experience

**DOI:** 10.3390/pediatric18040102

**Published:** 2026-08-03

**Authors:** Bartłomiej Kociński, Jowita Rosada-Kurasińska, Piotr Ładziński, Alicja Muszyńska, Diana Zawierucha, Robert Judek, Paweł R. Bednarek, Marcin Gładki, Alicja Bartkowska-Śniatkowska

**Affiliations:** 1 Department of Pediatric Cardiac Surgery, Poznan University of Medical Sciences, 61-701 Poznań, Poland; 2 Department of Pediatric Anesthesiology and Intensive Therapy, Poznan University of Medical Sciences, 61-701 Poznań, Poland; 3 Provincial Emergency Medical Services Station in Poznań, 60-346 Poznań, Poland

**Keywords:** PARDS, ECMO, respiratory failure, transportation

## Abstract

Objective: Extracorporeal Membrane Oxygenation (ECMO) has long been used in the treatment of acute respiratory and circulatory failure by providing time for damaged organs to recover. The aim of this study was to evaluate the safety and feasibility of interhospital transport of pediatric patients with acute respiratory failure who had undergone venovenous extracorporeal membrane oxygenation (VV ECMO) initiated at the referring facilities. Subjects and methods: Because of the critical condition of these patients, the high risk associated with transport, and the failure of conventional therapies, ECMO was initiated at the referring center. After cannulation, the patients were transported by ground ambulance to the Pediatric Intensive Care Unit in Poznań for further treatment. Results: Fourteen patients aged 2 months to 11 years with acute respiratory failure were transferred to our ECMO center. The mean time from decision to departure was 7.62 h, and the mean ICU stay before transfer was 4.14 days. The mean transport distance was 157.5 km. No mortality occurred during transport, and no serious adverse events were reported. Two technical complications were noted. Conclusions: Interhospital transport of pediatric patients on VV ECMO initiated at referring centers was feasible and safe, with favorable outcomes in patients who have exhausted conventional intensive care options. Effective collaboration between referring hospitals, ECMO centers, and emergency medical services was essential for optimal results.

## 1. Introduction

Extracorporeal Membrane Oxygenation (ECMO) has been used for many years in cardiac surgery centers to treat acute circulatory and respiratory failure. ECMO provides time for damaged organs to recover. Due to its specific indications and the complex management it requires in terms of both equipment and human resources, it is primarily performed in facilities that, in addition to appropriate technical infrastructure, have personnel trained and authorized to operate ECMO systems, often within cardiac surgery departments.

Ideally, patients should be transported to ECMO centers before their condition deteriorates to the point where conventional transport is no longer possible. However, in many cases, the clinical course is unpredictable, and patients’ condition can deteriorate rapidly. In such cases, rapid clinical deterioration may necessitate the initiation of ECMO at the referring hospital, followed by specialized interhospital transport. Although mobile ECMO programs have expanded in recent years, most published data originate from high-volume centers in Western Europe and North America, with limited evidence from Central and Eastern Europe [1]. In Poland, aside from our center, no other unit performs such transports, and there are no published reports from other Polish centers to date [2].

In Poland, pediatric patients with acute respiratory failure (ARF) are treated in Pediatric Intensive Care Units (ICUs) located in clinical hospitals, as well as in regional, municipal, and district hospitals across the country. Because of the critical condition of these patients, the excessive risk associated with transport, and the exhaustion of conventional therapies, the decision to initiate ECMO is sometimes made at the referring center. After cannulation, the patient is transported to our hospital for further treatment.

The ECMO technique was first used in 1972 by a team led by John Donald Hill at the California Pacific Medical Center [3]. Since then, its use in children has been primarily limited to the treatment of acute heart failure as extended extracorporeal circulatory support (reperfusion) after cardiac surgery or in neonatal respiratory failure. Depending on the method of connection between the ECMO circuit and the patient, the following types are distinguished [4]:

VA ECMO (venoarterial): blood is drawn from a vein, oxygenated, decarboxylated, and returned under pressure to an artery or aortic arch. In this configuration, it is used to support or temporarily replace the function of the heart and lungs.

VV ECMO: venovenous: blood is drawn from a vein and returned to the vena cava/right atrium. VV ECMO is used to support the respiratory system.

Indications for VV ECMO [5,6,7,8,9]:

ARF despite aggressive treatment in an intensive care setting.

a rapid course of the disease with a tendency to worsen.

a potentially reversible cause of ARF.

Contraindications for VV ECMO:

ARF as a terminal stage of a long-term illness

active bleeding into the central nervous system—CNS

no technical possibility of cannulation

lack of parental consent

expected non-survivability

Combes et al. designed the ECMO to Rescue Lung Injury in Severe ARDS (EOLIA) trial to determine the effect of early initiation of ECMO in patients with the most severe forms of acute respiratory distress syndrome (ARDS). Patients were considered for inclusion in the study if they met the American European Consensus Conference definition of ARDS, had been intubated and mechanically ventilated for less than 7 days, and, despite optimized ventilator settings, still fulfilled at least one of the predefined severity criteria. These criteria are now commonly used as inclusion thresholds for initiating ECMO in patients with severe ARDS. Criteria from the EOLIA trial include [8]:

PaO_2_/FiO_2_ < 50 mmHg >3 h or

PaO_2_/FiO_2_ < 80 mmHg >6 h

pH < 7.25 with PaCO_2_ > 60 mmHg

The aim of this study was to evaluate the safety and feasibility of interhospital transport of pediatric patients with acute respiratory failure supported with ECMO initiated at referring centers, as well as to describe transport organization, present clinical cases, and identify potential challenges encountered by the transport team.

## 2. Material and Methods

According to the rules of the Local Bioethical Committee of Poznan University of Medical Sciences, ethical approval is not required for retrospective documentation research and literature research, and therefore, no formal ethical approval was required (number of decision 418/25). All data processing was performed in accordance with the EU General Data Protection Regulation (GDPR) and applicable Polish legislation. Only pseudonymized datasets were used within the hospital, acting as the data controller, and no direct access to identifiable data was provided to journals or external reviewers.

We analyzed data (basic demographic variables, ECMO type, transfer details, and complications) from pediatric patients with acute respiratory distress syndrome (PARDS) who were initially treated in regional hospitals, cannulated for VV ECMO, and subsequently transported between January 2023 and December 2025 to the Karol Jonscher Clinical Hospital at Poznań University of Medical Sciences. Retrospective data were collected from our Hospital Information System (HIS). Transport complications were classified according to the risk category system published by Fletcher-Sandersjöö et al. [10]:

- Risk category I events: High risk for morbidity and mortality without response within seconds
- Risk category II events: High risk for morbidity and mortality with no response within minutes
- Risk category III events: Need for attention, with no risk to morbidity or mortality
- Risk category IV events: Low risk, needed to be noted

In this paper, we focus on the technical aspects of patient qualification, cannulation, circuit connection, and transport to our hospital.

### 2.1. Statistical Analysis

Descriptive statistics were used to summarize the demographic and clinical characteristics of the study participants. Categorical variables (e.g., sex) were presented as absolute numbers and percentages. Continuous variables (e.g., age, height, weight, distance, and time-related measures) were summarized using the mean and standard deviation (SD) to describe central tendency and variability, as well as the median and interquartile range (IQR, Q1–Q3) to account for potential skewness in the data. Minimum and maximum values (range) were also reported to illustrate the full data dispersion.

### 2.2. ECMO Procedure

The VV ECMO system was connected via peripheral cannulation using two veins: femoral-femoral, jugular-femoral, or jugular-jugular with a double-lumen cannula. Considering the internal vessel dimensions and the invasiveness of a sternotomy, the preferred cannulation approach in our patients was peripheral cannulation using an Avalon Elite^®^ double-lumen cannula (Bi-Caval Dual Lumen Catheter, Maquet Getinge Group, Rastatt, Germany).

The cannula size was selected according to the patient’s body weight and subsequently adjusted based on ultrasound assessment of the right internal jugular vein. The cannula is reinforced and includes a drainage lumen with distal suction openings positioned in the inferior vena cava (IVC) and proximal openings in the superior vena cava (SVC). The return lumen has a lateral opening located in the right atrium (RA), directing blood flow toward the tricuspid valve (TV). Connection to the ECMO circuit is achieved via a ¼-inch connector. The cannula does not have a dedicated skin fixation system. When organizing the cannulation and transport of a mechanically ventilated patient with VV ECMO support, the following factors must be considered:

cannulation is performed in a center with limited experience in VV ECMO support

the patient is critically ill, mechanically ventilated, often in the prone position

the circulation is stabilized with vasopressor support

adequate analgosedation is required

during transport, reperfusion injury, coagulation disorders, bleeding, hypovolemia, and inadvertent awakening may occur

potential early complications include critical damage to the vena cava or right atrium, air entrainment, or other mechanical failure of the ECMO system

When arranging a transport, we inform the referring facility of the following requirements:

operating room readiness

ultrasound with a linear probe

X-ray imaging

basic surgical instruments

blood products (min. 2 units of red blood cells, min. 1 unit of cryoprecipitate/10 kg, min. 2 units of FFP)

consent from a parent or legal guardian for high-risk therapy with an uncertain prognosis

We bring with us:

ECMO equipment approved for wheeled transport with a disposable kit

Avalon cannulas (the required size, plus spare cannulas of larger and smaller sizes)

introducers

equipment and medications necessary for routine intensive care during the first hours of VV ECMO: a ventilator, infusion pumps, monitoring equipment, and disposable pressure transducers

**Figure 1:**
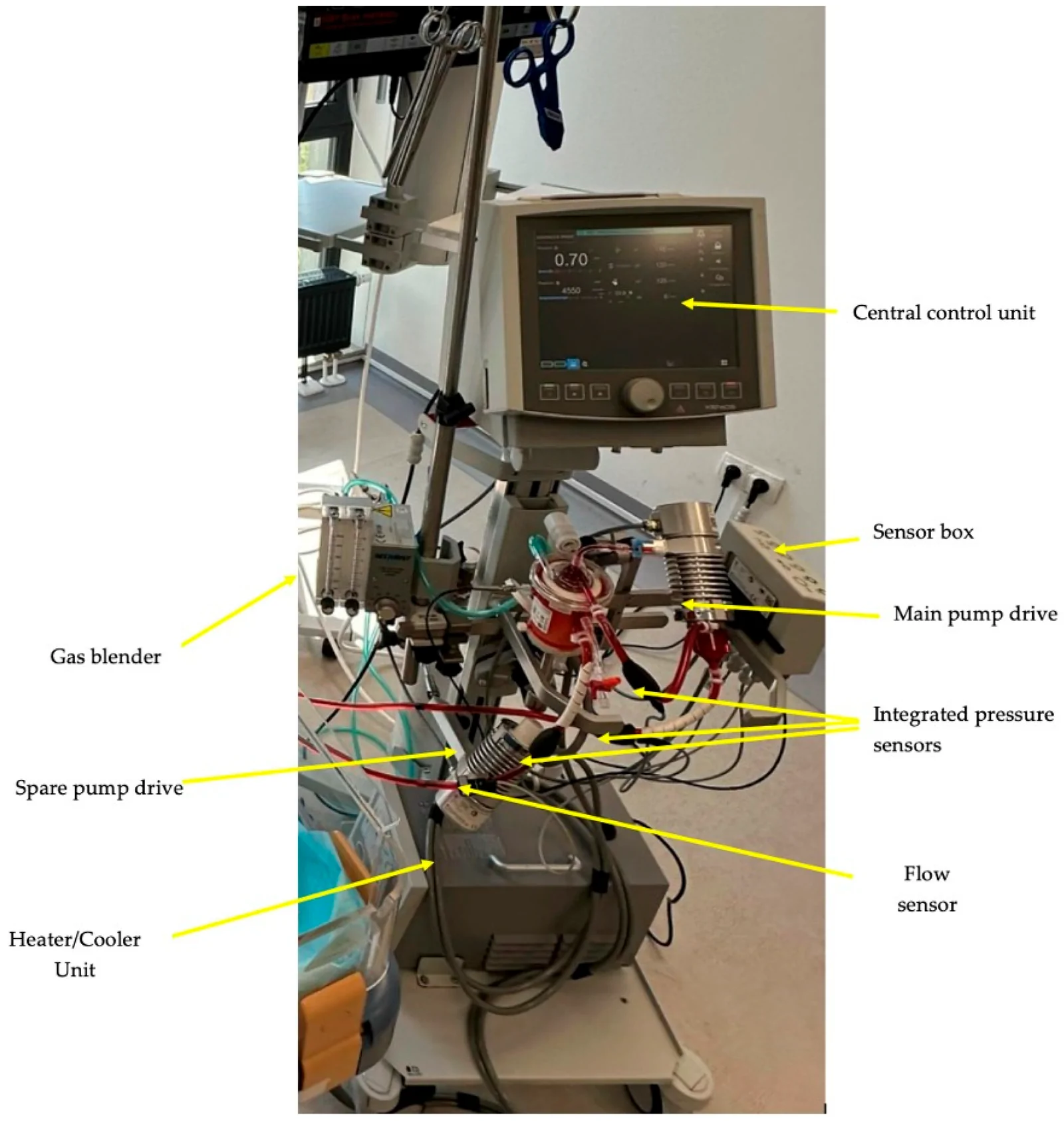
The Xenios console in the stationary configuration.

**Figure 2:**
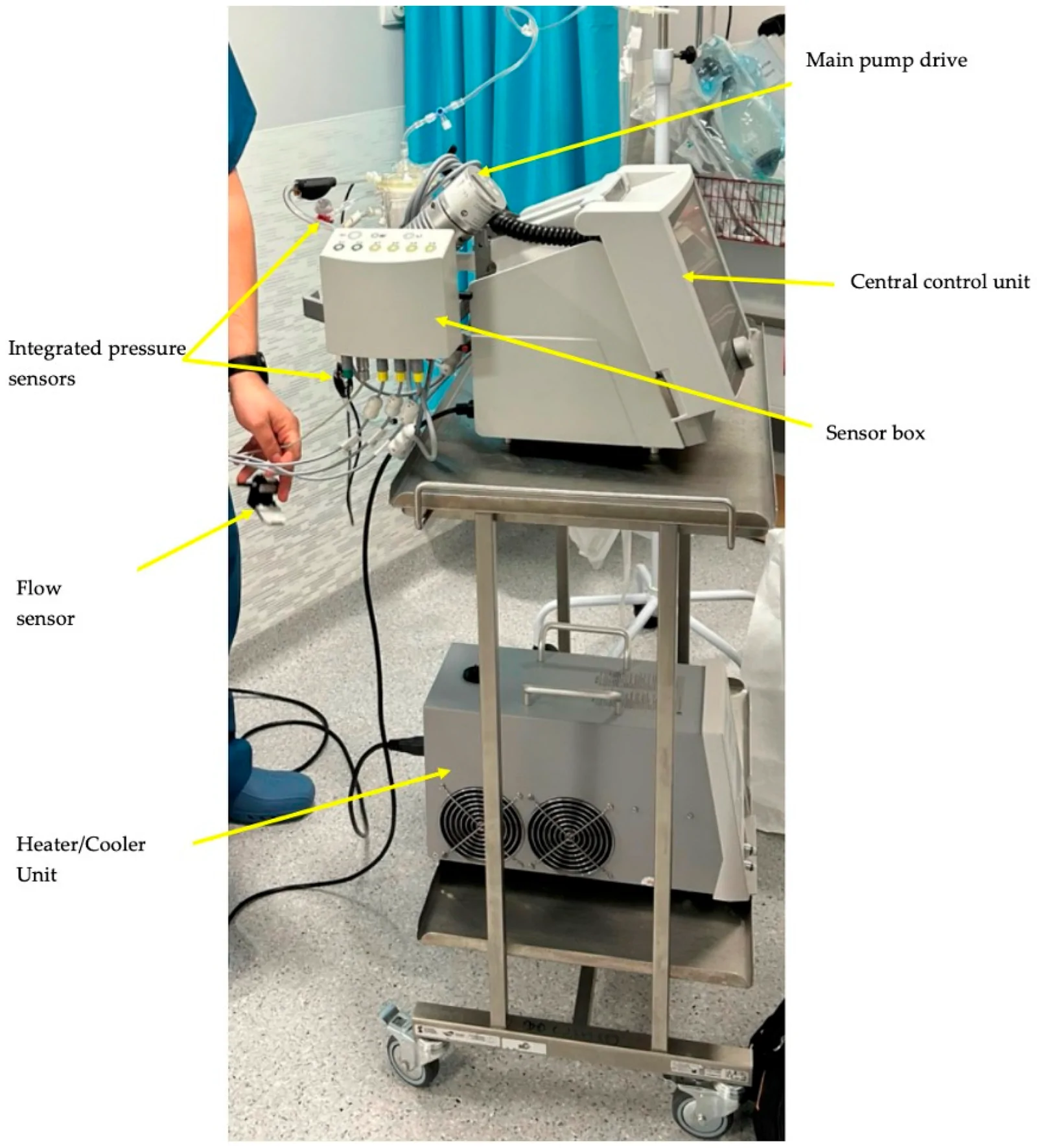
The Xenios console in the mobile configuration.

All transport equipment is stored in our department. Transports were performed with the Xenios ECMO system. The Xenios ECMO console is manufactured by Xenios AG, based in Heilbronn, Germany. Since 2016, Xenios AG has operated as a subsidiary of Fresenius Medical Care. The Xenios ECMO system is a versatile, multi-functional console capable of providing both pulmonary and cardiac support on a single platform. The system is designed for patients ranging from neonates to adults and has a modular architecture comprising several key components: a central control unit, a sensor box (interfacing the circuit sensors with the console), a primary pump drive (DP3), an identical battery-powered backup pump, an air-oxygen gas blender, and a heater-cooler unit. The backup pump allows a replacement circuit to be primed while the patient remains supported on the primary system. The gas blender offers two flow ranges: a pediatric mode for neonates and infants with flows up to 1 L/min, and a general pediatric mode with flows up to 10 L/min. The heater-cooler unit maintains patient blood temperature within a range of approximately 15 °C to 39 °C. The central unit operates on AC power and includes integrated batteries that ensure several hours of uninterrupted support in the event of a power failure. All sensors—including flow, pressure, bubble, and temperature sensors-provide non-invasive monitoring. The console also offers data-logging functionality with an on-screen alarm history for clinical review. An additional feature is a pulsed-flow mode integrated with electrocardiographic (ECG) monitoring, used exclusively during cardiac assist procedures. During in-hospital use, all components are mounted on a dedicated trolley. The Xenios console in the stationary configuration is shown in Figure 1, while the Xenios console in the mobile configuration is shown in Figure 2 (Figure 1 and Figure 2).

The Xenios system holds MultiSupport Ground certification in accordance with Regulation (EU) 2017/745 on Medical Devices (MDR), Annex IV, which permits its use for ground transport within European emergency medical services. To convert the Xenios console from a stationary to a mobile configuration, a compact holder is used to accommodate the main pump drive and sensor box. This holder attaches directly to the rear of the console, allowing the system to function as a portable “table unit” that can be carried or positioned in confined spaces. In this configuration, the pump drive, oxygenator, and monitor can be placed directly on the patient’s bed during transport. We use two types of preconfigured ECMO kits, compatible with the Xenios console and specifically certified for neonatal and pediatric use for up to 29 days. These kits are standard alternatives to Cardiohelp in centers that require dedicated pediatric certification and lower priming volumes. Each kit is coated with a multilayer heparin-albumin surface to minimize the risk of clotting and improve biocompatibility.

Compared with Cardiohelp’s smallest kit (approximately 570 mL total volume), the MiniLung petite has a markedly lower priming volume of 195 mL, which makes it a preferred option for neonates and small children in Poland.

The Emergency Response Team consists of at least three essential personnel [11]. Their responsibilities are displayed in Table 1.

driver: with a category C driving license—authorized to drive trucks in emergency mode

perfusionist: an operator of extracorporeal techniques, proficient in preparing and administering ECMO; during the journey, they supervise ECMO operations

anesthesiologist: a team leader experienced in vascular cannulation using ultrasound and X-ray imaging, and proficient in intensive care using extracorporeal techniques; during the journey, they are responsible for the patient’s general condition, ventilation, fluid and drug therapy, and monitor the power supply and operation of the equipment

assistance: a cardiac surgeon if difficulties with cannulation are anticipated; a physician and/or perfusionist trained in ECMO; the assisting team members participate in cannulation and system preparation

nurse: intensive therapy management

Avalon cannula implantation is performed under full aseptic conditions in the operating theater. The procedure begins with preparation of the operating room, including the availability of a fluoroscopy system (C-arm), an ultrasound device with a linear probe, an anesthesia workstation, and a complete set of surgical instruments. Blood products are prepared both for priming the ECMO circuit (filling volume) and for potential use in the event of massive bleeding, together with crystalloids for volume replacement. In addition to standard anesthetics used in ASA Physical Status 5 procedures, medications include a continuous adrenaline infusion, unfractionated heparin, and plasma-derived products such as Prothrombin Complex Concentrate (PCC), cryoprecipitate, or fibrinogen. Heparin at a dose of 50–100 IU/kg is administered at the time of guidewire insertion. After initiating ECMO and transferring the patient onto the stretcher, blood samples are collected for coagulation and morphology testing. The results are obtained during transport. Owing to the limited number of infusion pumps on board, continuous heparin infusion is initiated in the hospital after a control APTT test. Heparin is routinely used; no other anticoagulants are administered during transport.

Once the equipment is ready, a crucial step is a briefing with the operating room team to review the cannulation sequence and the ECMO pump activation protocol. Typical technical issues are discussed, together with the assistance expected from the operating team, bearing in mind that most of them have limited or no prior experience with ECMO procedures. Only after this debriefing and once all questions have been addressed, the patient is transported from the ICU to the operating room.

After the patient is placed supine on the operating table, the right internal jugular vein is punctured under direct ultrasound guidance using an out-of-plane technique [12]. The connection of the Avalon cannula is performed carefully to avoid air entrapment, as the ECMO circuit is a closed system without a venting option [13].

The first macroscopic sign of proper VV ECMO function is the color difference between the cannulas: a bright red color in the return line and a dark red color in the drainage line. This confirms that recirculation is not occurring within the superior or inferior vena cava or the right atrium. If a sector ultrasound probe is available, blood flow from the cannula to the right atrium and across the tricuspid valve can be visualized in the substernal view.

ECMO Blood Flow and Gas Exchange Management—Blood flow was typically initiated at 50 mL/kg/min and titrated towards a target of 150 mL/kg/min. However, pump flows above 100 mL/kg/min were often not achievable because of recirculation, markedly elevated pre- and post-oxygenator pressures (greater than 300 mmHg), and highly negative pre-pump pressures (below −100 mmHg). These limitations were managed by optimizing hemoglobin levels. Sweep gas flow was adjusted within a range of 0.5 to 2 times the extracorporeal blood-flow rate, according to arterial carbon dioxide levels, always with an FiO_2_ of 100%. Systemic anticoagulation was provided with unfractionated heparin, targeting a partial thromboplastin time (PTT) of 50–60 s.

From the moment VV ECMO is initiated in a child, the main sources of heat loss are the uninsulated cannulas, the oxygenator, and the pump. The equipment remains uninsulated to permit continuous visual monitoring for air bubbles, adherent thrombi, and possible unintended recirculation. Hypothermia is prevented by using an active heater-cooler unit that maintains appropriate thermoregulation. This device is powered by a 230 V AC supply. Unlike the ECMO and anesthesia systems, the heater-cooler is not battery-operated. During the transfer from the operating table to the transport stretcher and while the equipment is being secured for transport, the hospital oxygen and electrical supply remain connected.

## 3. Results

A total of 14 participants were included in our retrospective analysis. Most of the sample were male (78.6%, *n* = 11), while females accounted for 21.4% (*n* = 3). The median age was 16 months (interquartile range [IQR]: 13.25–33 months), with an age range from 2 to 132 months (0.17–11 years). The median height of participants was 76 cm (IQR: 72.5–83) and a range from 52 to 130 cm. The mean body weight was 10.9 kg (SD = 4.21), with a range between 4.3 and 20 kg. The mean time of departure information was 7.62 h (SD = 9.12), with a range from 1.5 to 36 h. The mean duration of ICU stay prior to transfer was 4.14 days (SD = 2.41), with a range of 1–10 days. The median transport distance was 151 km (IQR: 137–151) and a range between 5 and 520 km. Baseline characteristics of the study population are displayed in Table 2. There was no mortality during transfer, and no serious adverse events occurred. Two technical complications were encountered. All our patients at the beginning experienced an increase in arterial blood pressure (ABP), and it was necessary to reduce the dose of vasopressors if they had been previously used (continuation of the medication was discontinued in six cases, and in one case, vasodilator therapy was necessary). Diuresis was uncontrolled—access to a urinary reservoir was inaccessible. In one case, after receiving information (from the maternal hospital) about the INR value above 5, with accompanying fibrinogen (FBG) values below <60 mg/dL, APTT 60 s, PLT 60 × 103/μL and increasing bleeding around the cannula, prothrombin complex concentrate (PCC) preparations (Beriplex P/N manufactured by CSL Behring) were transfused and the appropriate persons at the home center were informed about the need to prepare cryoprecipitate preparations, which were transfused on board the ambulance before preparation for disembarking the patient.

In three cases, patients required additional doses of unfractionated heparin due to non-therapeutic APTT values. Fluid therapy, along with additional boluses due to relative hypovolemia, was administered to all our patients. Three patients required tracheobronchial drainage. These procedures were performed while the ambulance was in motion, with seat belts fastened, by all team members, without the need to stop the vehicle.

One technical problem occurred during transport, classified as Category III risk—no immediate risk of morbidity or mortality but requiring attention. During one transport, an increased loss of oxygen (O_2_) pressure in the main cylinders was observed in one patient. The patient required VV ECMO with a sweep of 1.1 L/min, FiO_2_ 1.0, and a pump output of 1600 mL/min. He was additionally ventilated with a HAMILTON C1 ventilator in CMV mode, FiO_2_ 1.0, Tv 150 mL, and BR 45/min. Due to O_2_ consumption significantly exceeding the expected values, the ambulance was stopped. No leak was found, but the cylinders were cold and frosted. The ambulance was switched to an emergency ventilator, and the journey continued. Due to the oxygen supply approaching critical levels, the Poznań Emergency Medical Services Center (WSPR) and the Emergency Communication Center were informed of the need for additional supplies. The team was directed to the nearest hospital to replenish the oxygen supply. After replenishment, the journey continued. The transport concluded with the patient being transferred in stable condition for further treatment at his home facility.

## 4. Discussion

Bartlett et al. were the first to report cannulating patients at peripheral hospitals before ECMO transport [14]. The decision to initiate the VV ECMO procedure was made by the intensive care physician at the referring center. The decision to proceed with cannulation is then taken jointly, based on ELSO guidelines and the exhaustion of other therapeutic options. The final decision is made by the anesthesiologist of the ECMO team. Once a decision is reached, all team members are informed. At the same time, we verify the availability of an ambulance and a driver. After the entire team has been organized, we contact the referring hospital again.

The qualifying criteria included prior use of all available intensive treatment modalities for ARF, lack of improvement or further deterioration in gas exchange parameters, worsening overall clinical condition, and exhaustion of conventional therapeutic options. The qualification process is extremely difficult due to the following factors:

lack of experience with ECMO resulting from the unavailability of this procedure in centers without cardiac surgery facilities

lack of clear guidelines and inclusion criteria, lack of dedicated centers on duty, particularly for pediatric patients

lack of a regulated legal status for a team operating in one hospital within another center

distance from the ECMO center

lack of constant on-call ECMO team

In the cases described, from the moment initial information was received, continuous communication was maintained with the referring center. The decision to initiate ECMO was made gradually, based on the patient’s overall condition, laboratory results, ongoing therapy, and the dynamic course of the disease. The mean length of intensive care stay in our study was 4.14 days, whereas Belda Hofheinz et al. reported a mean stay of only 2 days. This difference highlights how challenging decision-making can be in centers with limited experience in ECMO therapy [15].

The clinic’s team does not operate on a regular ECMO on-call schedule. Data indicate that when ECMO-supported transport is conducted by an experienced team, severe complications are not associated with increased morbidity or mortality [16]. When a transport request is submitted to the ECMO transport coordinator, a specialized team is assembled, consisting of 1–2 anesthesiologists, 1–2 perfusionists, a nurse and a cardiac surgeon if complications during cannulation are anticipated, in addition to an ambulance driver. Padalino et al. described distinct roles for each team member involved in ECMO management. The same structured approach applies in our setting [11]. We also emphasize the need for extensive team experience, as highlighted by Fletcher-Sandersjöö. He demonstrated that a mobile ECMO program requires a highly skilled transport team available on-call, capable of evaluating patients at the referring hospital, initiating cannulation or starting ECMO therapy on-site, and safely transferring the patient to a specialized ECMO center [10].

After VV ECMO implantation and initiation, the patient often remains hemodynamically unstable, requiring parenteral catecholamines and gradual adaptation to new homeostatic conditions. During the early phase of VV ECMO therapy, it is frequently necessary to adjust the dose or change the type of catecholamines, administer coagulation factors (as cannulation may be associated with significant bleeding), and continuously titrate analgesia, sedation, and fluid management. These requirements highlight the importance of reliable intravenous access and continuous monitoring, including ECG and peripheral oxygen saturation (SpO_2_) assessment [17].

In all VV ECMO cases managed at our center, the extracorporeal life support (ECLS) system demonstrated sensitivity to patient repositioning during the first few days of therapy. Positional changes may cause venous blood redistribution and relative hypovolemia in the inferior or superior vena cava and cannula drainage ports, leading to transient pump dysfunction or even pump arrest. As the patient stabilizes, this sensitivity diminishes, enabling optimal care and, for example, safe prone positioning for ventilation. Similar physiological findings related to VV ECMO have also been reported by Tomarchio et al. in their research, where they reviewed the complex physiology of patients supported with VV ECMO [18].

Another important factor is the availability of an ambulance from the Provincial Emergency Medical Services Station in Poznań, along with a driver holding a category C license, as the ambulance is classified as a truck due to its weight. The choice of ground transport is determined by the patient’s condition and the need to provide continuous intensive care during the critical initial hours of VV ECMO support. In the case of air transport using the EC135 helicopter from the Polish Medical Air Rescue (LPR) fleet, the cabin accommodates only two medical crew members: a physician and a paramedic [19]. Immediate flight interruption is not feasible due to the lack of space for a third essential team member, the perfusionist. In contrast, air transport with the Piaggio P180 aircraft operated by the Polish Medical Air Rescue (LPR) allows for the full team, although immediate flight termination is still not an option [20].

Due to severely limited venous drainage (caused by the cannula in the superior vena cava and elevated intrathoracic pressure), the Fowler position should be avoided to prevent increases in intracranial pressure during gradual reoxygenation and decarboxylation. The selected mode of transport should provide the most stable surface possible, without tilting in any direction. From the perspective of helicopter flight mechanics, the pilot controls direction by altering the aircraft’s tilt, a simplified but relevant principle; thus, a fully stable surface cannot be ensured, potentially exacerbating intracranial pressure or relative hypovolemia.

Ground transport by ambulance therefore offers a clear advantage. According to ELSO guidelines, ground transport is recommended for distances up to 400 km (250–300 miles), and helicopter transport for distances up to 650 km (300–400 miles) [21]. Our Xenios 1.0 system includes pediatric kits but is not certified for air transport. The Cardiohelp System, currently certified for air operation, lacks dedicated pediatric components [22].

Considering all these factors, our experience with out-of-center cannulation, the need for intensive care during the initial hours of ECMO support, and the comparable survival rates reported for VV ECMO transport in adults, we consistently select ground ambulance transport [23]. At present, despite the absence of a statistically significant advantage of ground over air transport, we find no justification to modify our approach within Poland [24]. We are aware that these are only our experiences and the existing evidence in the literature contains reports on the safe air transport of children with ECMO support. The available literature clearly indicates that air transport can also be performed safely, as demonstrated by Kendirli in his study [25]. He concluded that mobile pediatric extracorporeal membrane oxygenation (ECMO) teams can ensure safe air and ground transport for high-risk patients. However, Fletcher-Sandersjöö stated that transport by fixed-wing aircraft was linked to an increased risk of serious transport-related complications [10].

During transport, oxygen pressure gauges in the main supply cylinders and device charge indicators were checked at 15 min intervals, confirming the proper operation of the 230 V system. The patient’s condition was monitored by monitors: SpO_2_, ECG, IABP, temperature, and the ECMO pump control panel. Continuous monitoring of the patient during transport is essential and has been emphasized in studies on ECMO patient transport, including those by Fletcher-Sandersjöö and Kendirli [10,25]. The following are displayed: pump speed (rpm), flow (L/min), proximal pressure (between the venous cannula and the pump), and distal pressure (between the oxygenator and the arterial cannula). Sweep (gas flow through the oxygenator) was regulated by a flow regulator. 100% oxygen concentration was used during transport.

## 5. Conclusions

VV ECMO initiated at the referring hospital can be transported safely, achieving satisfactory survival rates (79%) in patients for whom standard intensive care options have been exhausted

ground transport available in Poland enables the provision of full intensive care on board a properly equipped ambulance, provided it contains appropriate medical equipment and is staffed by a specialized team including an anesthesiologist, a cardiac surgeon, and a perfusionist

collaboration between multiple services: hospitals, clinical centers, and emergency medical services results in highly favorable outcomes

of the fourteen transports performed following VV ECMO initiation, eleven children were ultimately discharged home in good condition

## Figures and Tables

**Table 1:** Personnel and roles of the ECMO team.

Personnel	Before Departure	Departure	Cannulation	Transport of the Patient
anesthesiologist/intensive care physician	patient qualification, control of taken medications and equipment	contact with the referring center	cannula implantation, connection to ECMO	intensive care management of critically ill patients
perfusionist	preparation of the pump, set, cannulas, implantation kit		preparation of the ECMO device for connection, start-up	supervision of the ECMO device, control of AC power and O_2_ supply
assistant	preparation of medications and equipment		assistance with implantation, assistance to the operating theater staff of the referring center	patient management during transfer, including monitoring, drug preparation
pediatric cardiac surgeon			patient management during surgical procedure, surgical positioning of the cannulas	patient management during transfer, including monitoring, drug preparation
nurse	preparation of medications and equipment			patient management during transfer, including monitoring, drug preparation
driver	securing oxygen supply, first aid bags, checking the 12/230 V converter, checking batteries, fuel	driving a priority vehicle	parking, heater activated	driving a priority vehicle taking particular care to avoid shocks and sudden changes in speed and direction

**Table 2:** Demographic and clinical characteristics.

Variable	Parameter	Overall (*n* = 14)
sex	female	21.4% (*n* = 3)
	male	78.6% (*n* = 11)
age [months]	*n*	14
	median (Q1–Q3)	16 (13.25–33)
	range	2–132
height [cm]	*n*	14
	median (Q1–Q3)	76 (72.5–83)
	range	52–130
weight [kg]	*n*	14
	mean (SD)	10.9 (4.21)
	range	4.3–20
information-departure [h]	*n*	14
	mean (SD)	7.62 (9.12)
ICU before [days]	*n*	14
	mean (SD)	4.14 (2.41)
distance [km]	*n*	14
	median (Q1–Q3)	151 (137–151)
	range	5–520

## Data Availability

The datasets used and/or analyzed during the current study are available from the corresponding authors on reasonable request.

## Abbreviations

ECMO	extracorporeal membrane oxygenation
ELSO	Extracorporeal Life Support Organization
ECLS	Extracorporeal Life Support
VV	venovenous
VA	venoarterial
EOLIA	the ECMO to Rescue Lung Injury in Severe ARDS
ARDS	acute respiratory distress syndrome
ARF	acute respiratory failure
ICU	Intensive Care Unit
PARDS	pediatric acute respiratory distress syndrome
CNS	central nervous system
ASA	scale of American Society of Anesthesiologists
APTT	Activated Partial Thromboplastin Time
LPR	the Polish Medical Air Rescue
PCC	Prothrombin Complex Concentrate

## References

[1] Rosada-Kurasińska J., Kociński B., Wiernik A., Gładki M., Puślecki M., Ładziński P., Ogino M.T., Bartkowska-Śniatkowska A. (2026). Pediatric Extracorporeal Membrane Oxygenation (ECMO) Transport Safety-Regional and National Experiences and Literature Review. J. Clin. Med..

[2] Rosada-Kurasińska J., Kociński B., Ogino M.T., Wiernik A., Gładki M., Puślecki M., Bartkowska-Śniatkowska A. (2026). Mobile versus in-center ECMO initiation in pediatric patients: A comparative outcome study. Anaesth. Crit. Care Pain Med..

[3] Hill J.D., O’Brien T.G., Murray J.J., Dontigny L., Bramson M.L., Osborn J.J., Gerbode F. (1972). Prolonged extracorporeal oxygenation for acute post-traumatic respiratory failure (shock-lung syndrome). Use of the Bramson membrane lung. N. Engl. J. Med..

[4] Conrad S.A., Broman L.M., Taccone F.S., Lorusso R., Malfertheiner M.V., Pappalardo F., Di Nardo M., Belliato M., Grazioli L., Barbaro R.P. (2018). The Extracorporeal Life Support Organization Maastricht Treaty for Nomenclature in Extracorporeal Life Support. A Position Paper of the Extracorporeal Life Support Organization. Am. J. Respir. Crit. Care Med..

[5] Mang S., Karagiannidis C., Lepper P.M. (2023). Wenn maschinelle Beatmung nicht mehr ausreicht—Venovenöse extrakorporale Membranoxygenierung [When mechanical ventilation fails—Venovenous extracorporeal membrane oxygenation]. Inn. Med..

[6] Vyas A., Bishop M.A. (2025). Extracorporeal Membrane Oxygenation in Adults. StatPearls [Internet].

[7] Banfi C., Pozzi M., Siegenthaler N., Brunner M.-E., Tassaux D., Obadia J.-F., Bendjelid K., Giraud R. (2016). Veno-venous extracorporeal membrane oxygenation: Cannulation techniques. J. Thorac. Dis..

[8] Langer T., Malbrain M.L., Van Regenmortel N. (2020). Hypotonic or isotonic maintenance fluids for paediatric patients: The never-ending story. Anaesthesiol. Intensive Ther..

[9] Combes A., Hajage D., Capellier G., Demoule A., Lavoué S., Guervilly C., Da Silva D., Zafrani L., Tirot P., Veber B. (2018). Extracorporeal membrane oxygenation for severe acute respiratory distress syndrome. N. Engl. J. Med..

[10] Fletcher-Sandersjöö A., Frenckner B., Broman M. (2019). A Single-Center Experience of 900 Interhospital Transports on Extracorporeal Membrane Oxygenation. Ann. Thorac. Surg..

[11] Padalino M.A., Doglioni N., Nardo D., Baraldi E., Vida V.L., Trevisanuto D. (2021). The “Hub and Spoke” (HandS) ECMO for “resuscitating” neonates with respiratory life-threatening conditions. Children.

[12] Rosario D.C., Ambati S. (2025). Extracorporeal Membrane Oxygenation in Children. StatPearls [Internet].

[13] Kilic O., Gultekin Y., Yazici S. (2020). The impact of intravenous fluid therapy on acid-base status of critically ill adults: A Stewart approach-based perspective. Int. J. Nephrol. Renovasc. Dis..

[14] Bartlett R.H., Gazzaniga A.B., Fong S.W., Jefferies M.R., Roohk H.V., Haiduc N. (1977). Extracorporeal membrane oxygenator support for cardiopulmonary failure. Experience in 28 cases. J. Thorac. Cardiovasc. Surg..

[15] Belda Hofheinz S., López Fernández E., García Torres E., Dachary J.A., Boni L., Llopis I.T., Gámez R.O., Rodríguez L.C., Bravo M.M., Gámez S.L. (2024). Primary neonatal and pediatric ECMO transport: First experience in Spain. Perfusion.

[16] Barrigoto C., Fortuna P., Silva P.E., Bento L. (2024). Complications during transport of adult patients on extracorporeal membrane oxygenation. Perfusion.

[17] Rosada-Kurasinska J., Bartkowska-Śniatkowska A., Zielińska M., Mikaszewska-Sokolewicz M., Kociński B., Jaśkiewicz R., Pagowska-Klimek I. (2026). Consensus statement of the Paediatric Anaesthesiology and Intensive Therapy Section of the Polish Society of Anaesthesiology and Intensive Therapy on the use of VV ECMO in paediatric patients for the treatment of acute respiratory failure. Anaesthesiol. Intensive Ther..

[18] Tomarchio E., Momigliano F., Giosa L., Collins P.D., Barrett N.A., Camporota L. (2024). The intricate physiology of veno-venous extracorporeal membrane oxygenation: An overview for clinicians. Perfusion.

[19] Polish Medical Air Rescue (LPR). https://www.lpr.com.pl/o-nas/flota/ec135-h135-p3.

[20] Polish Medical Air Rescue (LPR) Piaggio P180 Helicopter. https://www.lpr.com.pl/o-nas/flota/piaggio-p180.

[21] MacLaren G., Brodie D., Lorusso R., Peek G., Thiagarajan R., Vercaemst L. (2022). Extracorporeal Life Support: The ELSO Red Book.

[22] Hermann A., Schellongowski P., Robak O., Buchtele N., Nagler B., Müller M., Staudinger T. (2024). Our approach for out-of-center initiation of extracorporeal membrane oxygenation and subsequent interhospital transport. Wien. Klin. Wochenschr..

[23] Rypulak E., Szczukocka M., Czarnik T. (2024). Utilisation and outcomes of a mobile (ambulance and air transport) venovenous extracorporeal membrane oxygenation (VV-ECMO) program in Poland during the COVID-19 pandemic—A retrospective, two-centres, case-series study. Anaesthesiol. Intensive Ther..

[24] Smith G.C., Pell J.P. (2003). Parachute use to prevent death and major trauma related to gravitational challenge: Systematic review of randomised controlled trials. BMJ.

[25] Kendirli T., Kahveci F., Özcan S., Botan E., Sarıcaoğlu C., Hasde A.İ., Cakici M., Ucar T., Eyileten Z., Tutar E. (2022). Interhospital Aircraft/Ground Extracorporeal Membrane Oxygenation Transportation by a Mobile Extracorporeal Membrane Oxygenation Team: First Turkish Pediatric Case Series. Turk. Arch. Pediatr..

